# Deposition Contribution Rates and Simulation Model Refinement for Polysilicon Films Deposited by Large-Sized Tubular Low-Pressure Chemical Vapor Deposition Reactors

**DOI:** 10.3390/ma17235952

**Published:** 2024-12-05

**Authors:** Jicheng Zhou, Jianyong Zhan, Bowen Lv, Yan Guo, Bingchun Jiang

**Affiliations:** 1 School of Mechanical and Electrical Engineering, Guangdong University of Science and Technology, Dongguan 523083, China; 2 School of Energy Science and Engineering, Central South University, Changsha 410083, China

**Keywords:** TOPCon solar cells, LPCVD, polysilicon films, multi-physics coupling, simulation, model refinement

## Abstract

Tunnel oxide passivating contact cells have become the mainstream form of high-performance photovoltaic cells; however, the key factor restricting the further improvement of tunnel oxide passivating contact cell performance lies in the deposition process technology of high-quality polysilicon films. The experimental optimization cost for the deposition of large-sized polysilicon films in low-pressure chemical vapor deposition reactors is enormous when conducted in the temperature range of 800–950 K; hence, the necessity to develop effective computer simulation models becomes urgent. In recent years, our research group has conducted two-dimensional simulation research on large-sized, low-pressure chemical vapor deposition. This article focuses on analyzing the influence of gas-phase chemical reactions on the contribution rate of polysilicon film deposition under a mixed atmosphere of H_2_ and SiH_4_. The findings indicate that when using SiH_4_ as the precursor reactants with a gas pressure not exceeding 100 Pa, SiH_4_ contributes more than 99.6% to the deposition of polysilicon films, while the contribution rate of intermediates from chemical reactions to film deposition is less than 0.5% with 860–900 K. The influence of temperature on the contribution rate of gas-phase intermediates is negligible. It is found that simulating complex multi-step chemical reactions is highly resource-intensive, making it difficult to achieve the three-dimensional simulations of large-sized tubular LPCVD reactors. Based on the in-depth analysis of the mechanism and simulation results, a simplified model neglecting the complex multi-step chemical reaction process has been proposed. Through employing this refined and simplified model, the two-dimensional simulation of the polysilicon thin films deposition process in the large-sized tubular low pressure chemical vapor deposition reactor will become more effective and resource efficient.

## 1. Introduction

Tunnel oxide passivating contact (TOPCon) cells require the deposition of a large-area polysilicon film, which is generally fabricated through the high-temperature pyrolysis of SiH_4_. The non-uniformity of the film has a significant impact on photoelectric conversion efficiency [1,2,3,4,5]. Low-pressure chemical vapor deposition (LPCVD) is an important method for preparing polysilicon films, with low energy consumption, vertically packed silicon wafers, and high yield [6]. In theory, due to the use of low-pressure processes, the molecular free path increases, and the deposition uniformity is better than that of atmospheric pressure processes [7,8,9,10].

However, the fabrication of polysilicon films by the LPCVD method is an extremely complex, multi-step chemical reaction process, involving more than 20 distinct chemical reactions [11,12,13,14,15,16]. Kinoshita S. et al. [17] constructed a two-dimensional simulation model for submicron hole silicon LPCVD and found that two intermediates, Si_2_H_6_ and SiH_2_, deteriorate the uniformity of film deposition. Kleijn C. R. [18] established a mathematical model and a chemistry model, including fluid flow and transport dynamics and reaction mechanism. He believes that although there are many precursors that can affect the growth rate of polysilicon films, it is appropriate to use SiH_4_ Si_2_H_6_, Si_2_H_4_, and Si_3_H_8_ to predict the growth rate. The reference [18] has been extensively cited by researchers studying the growth of polysilicon films by LPCVD and is referred to as the Kleijn Model. Shimizu R. et al. [19] established a theoretical optimization model for LPCVD polysilicon film deposition on a 6-inch wafer, discovering that the two intermediates, Si_2_H_6_ and SiH_2_, due to their high sticking probabilities, are unable to reach the center of the wafer, and thus worsen the film’s uniformity. The key to improving the uniformity of polysilicon film distribution is suppressing SiH_2_ and Si_2_H_6_. Houf W. G. et al. [20] proposed a SiH_4_ reaction kinetics model and predicted the growth rate of polysilicon films. They gave a fundamental description of the chemical kinetics of silane, which is based on molecular beam measurements of the reactive sticking coefficient of silane on silicon. Ho P et al. [21] simplified the previous gas-phase reaction mechanism. They compared in detail predictions from a CFD simulation model of rotating CVD from silane and disilane that treats the fluid flow coupled to gas-phase and gas–surface chemistry.

There have been many achievements in the research on elements such as reaction kinetics, theoretical optimization models, and the role of intermediates [10,11,17,18,19,20,21,22,23,24,25]. However, there is little research on the contribution rate issue of large-sized polysilicon films, and no relevant reports on three-dimensional simulation research regarding polysilicon film deposition have been found. Therefore, modeling refinement and 3D simulation are crucial for improving the uniformity and performance of polysilicon films in large-scale production environments.

TOPCon cells have high theoretical photoelectric conversion efficiency and high compatibility with Passivated Emitter and Rear Cells (PERCs) in their mass production process and have been rapidly mass-produced in recent years. Currently, TOPCon cells account for more than 60%. According to some sources, TOPCon cells will continue to dominate for the next five years [25]. The size of mass-produced silicon wafers for cells is increasing, and the loading capacity of a silicon wafer in a single tube is also increasing. This results in larger diameters of LPCVD reaction chambers and longer tubular reactors. The large size leads to an increase in the non-uniformity of the physical field inside the reaction chamber [26,27,28,29,30], further deteriorating the issue of film uniformity [31,32,33]. Consequently, the challenges in achieving accurate simulations are amplified, making it crucial to develop advanced modeling techniques and more precise calibration methods to address these complexities

In response to the large-sized LPCVD uniformity issues, the demand for computational simulation resources has shown an exponential increase. Simulating all the multi-step chemical reaction mechanisms has become increasingly difficult [34,35,36].

As mentioned above, although numerous references have explored the simulation of polysilicon film deposition [16,17,19,20,21], these studies have primarily focused on small laboratory samples. Due to the resource-intensive nature of multi-step chemical reactions in the simulation process, the simulations have been limited to two dimensions.

Understanding the kinetics and the mechanisms of SiH_4_ pyrolysis has always been the goal pursued by peers in this field [14,15,16,17,19,20,21]. The establishment of efficient simulation methods for large-sized polysilicon films within tubular LPCVD reactors has become increasingly urgent.

For the large-sized tubular LPCVD reactor we chose, we initially attempted to establish a three-dimensional simulation scheme. However, it took a very long time and was difficult to converge. We had to give up as we found that multi-step reaction simulation would take a lot of time, which is the main reason for the failure of three-dimensional modeling of large-sized polysilicon film deposition systems. A two-dimensional multi-physics field model for computer simulation was constructed by our research group [6].

This paper is also based on the software COMSOL Multiphysics v.5.6 [37], focusing on the analysis of the contribution rate of reactants and intermediates to film deposition in the two-dimensional simulation model. We also hope to expand the effective reaction space for depositing polysilicon films. Moreover, we envision that if the simulation model of polysilicon film deposition processes can be refined, it becomes possible to establish a three-dimensional simulation model. We have proposed the concept of the contribution rate of polysilicon film deposition. The contribution rate of polysilicon films, film deposition process parameters, and the influence of intermediates are key points explored. These works have important fundamental support for establishing new and efficient three-dimensional simulation schemes.

## 2. Experimental Issues and Simulation Scheme

**Figure 1:**
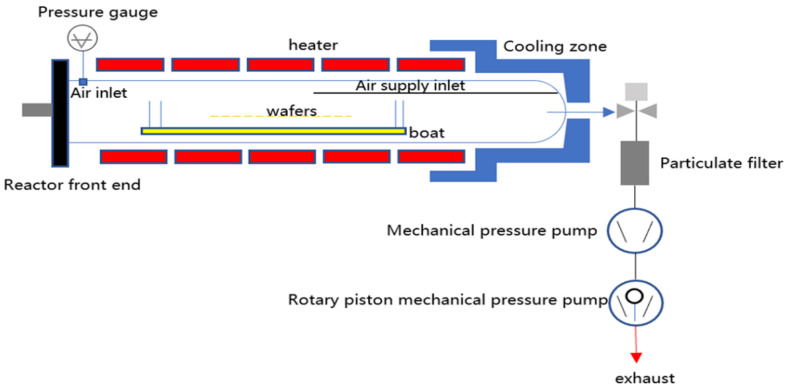
The schematic diagram of the tubular LPCVD reactor. This reactor structure and parameters originate from Hunan Red Solar Photoelectricity Science and Technology Co., Ltd., Changsha, China.

**Figure 2:**
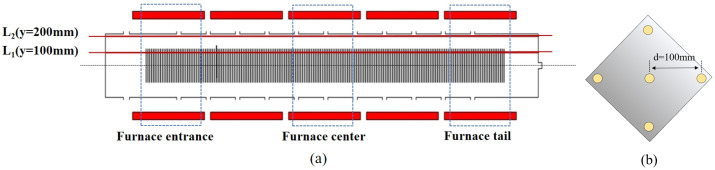
(**a**) The schematic diagram of five temperature zones and wafer vertical placement. Except for buffer wafers, 1200 silicon wafers are uniformly arranged in the five temperature zones; in each temperature zone, there are 240 silicon wafers. The lengths of the middle three temperature zones are 480 mm; the two extremities are 300 mm longer. In the outermost space of the extremities, there are no silicon wafers. The test silicon wafer samples were taken from the 1st to 3rd, 119th to 121st, and 238th to 240th wafers in the leftmost, middle, and rightmost temperature zones, respectively. (**b**) The sketch map of polysilicon thickness measurement points for each wafer.

The tubular LPCVD reactor was a ϕ420 mm cylindrical oven, had an operating pressure of 20~300 Pa, and a temperature control range of 673 K to 973 K, as shown in Figure 1. The reaction chamber was divided into 5 temperature zones. The three temperature zones in the middle had a length of 480 mm. The temperature zones at both ends had a length of 780 mm. The length of the temperature zones at the two extremities was 300 mm longer than that of the temperature zones in the middle. The extra space was not used for placing silicon wafers, which means there was a 300 mm buffer zone after the two extremities. The size of the square monocrystalline silicon wafer was 182 mm × 182 mm, and the wafer thickness was 175 μm. The 1200 slices of monocrystalline silicon wafers were symmetrically vertically placed and densely lined up in the middle of the chamber, as shown in Figure 2a. The spacing between the vertically placed wafers was 2 mm. All experiments were conducted in a steady state.

In this paper, the 2D steady-state simulation of polysilicon film deposition was based on the pre-programmed heat transfer module, flow module, and chemical reaction module in COMSOL [37]. The boundary conditions and hypothesis were as follows: (1) Considering the expansion of the gas mixture due to heating during transport, the gas mixture was recognized as an uncompressible gas (Ma < 0.3). (2) The inlet temperature and temperature zone temperatures were recognized as constant values, the temperatures of the five zones were set to 885 K, 885 K, 884 K, 883 K, and 882 K (along the direction of the gas flow), and the inlet temperature was set to 293.15 K. (3) With low Rayleigh number airflow in the chamber, the laminar flow model was selected for calculation. (4) The equipment used an excess of pure silane as the gas source, with a total inlet flow of 590 sccm and an outlet pressure of 26 Pa. (5) The inner wall of the equipment had a blocking friction effect on the gas flow, and the wall boundary conditions were set as no-slip conditions. (6) The temperature of the chamber cover and its nearby environment was set to 363 K.

In this article, the maximum flow velocity of gas impacting the first monocrystalline silicon wafer was around 1.7 m/s, the dynamic viscosity of silane was about 0.00002 N·s/m^2^, the density was about 0.0002 kg/m^3^, and the characteristic length was 0.2873 m. The calculated Reynolds number was 4.375. Since the calculated value of the Reynolds number was much smaller than 2300, it was reasonable to assume that the flow field was laminar.

The division of grids requires the consideration of multiple aspects. Furthermore, the surface reaction region is the main research object of this article [6]. Due to the complex flow field distribution, a boundary layer grid was set up on the front region of the first silicon wafer. Moreover, because the airflow velocity between the middle silicon wafers was almost zero and the physical properties of the airflow changed very weakly, this region was divided by a triangular grid. For boundaries with heat exchange or with temperature gradients, a dense grid division was used to obtain a more accurate temperature field distribution.

To obtain accurate calculation results and minimize the calculation time of the simulation, L_2_ (y = 200 mm, in Figure 2a) was defined as the monitoring line. The simulation data of airflow velocity were taken from the points on L_2_. Line L_2_ was selected as the observation object.

Different grid sizes seriously impact the calculation results of airflow velocity. In this study, four groups of grids were used for grid independence study. When the grid size (mesh number) reached 340,578, the improvement of the accuracy of airflow velocity calculation was very limited.

The deposition process of LPCVD polysilicon films involves many steps and various kinds of chemical reactions, such as SiH_4_ molecule transfer, SiH_4_ adsorption, SiH_4_ pyrolysis, pyrolysis intermediates adsorption, homogeneous reactions between the gas matter, and diffusion from the silicon surface to the bulk. All chemical reactions can be divided into two categories, homogeneous reactions and heterogeneous reactions. After being introduced into the reaction chamber, SiH_4_ first pyrolyzes into silylene (SiH_2_) and hydrogen gas. During the deposition process, SiH_4_ and SiH_2_ react to generate multifarious intermediates, as shown in Table 1. These intermediates can also adsorb onto the monocrystalline silicon wafer surface and undergo chemical reactions, ultimately forming a polysilicon film. Table 1 presents the homogeneous gas-phase reactions and their reaction rate constants [36].

The low-pressure gas stream of the precursor at room temperature will continuously heat up and expand the gas volume after entering the tubular LPCVD reactor. This low-pressure gas can be assumed to be an incompressible fluid. The gas flow in the chamber can be assumed to be laminar. Under these two assumptions, it was appropriate to use a laminar flow model to establish the basic simulation equations [14,15,16,37]:

(1) $$\frac{\partial \rho }{\partial t}+\nabla \cdot \left(\rho u\right)=0,$$

(2) $$\frac{\delta }{\delta t}(\rho \omega_{k})+\nabla \cdot (\rho \omega_{k}u_{k})=R_{k}M_{k}\;(k=1,\ldots n),$$

(3) $$\frac{\delta }{\delta t}(\sum_{k=1}^{n}\rho \omega_{k}H_{k})+\nabla \cdot (-\lambda \nabla T_{g}+\sum_{k=1}^{n}\rho \omega_{k}H_{k}u_{k})=0,$$

(4) $$\frac{\delta }{\delta t}[\Gamma_{n}{Ζ}_{k}(n)]=s_{k}\sigma_{k}(n)\;(k=1,\ldots m).$$

where ρ is the density of the gas mixture, u is the average velocity at this location, $\omega_{k}$ is the mass fraction of substance k, $u_{k}$ is the average velocity of substance k at this location, $R_{k}$ is the homogeneous gas-phase reaction rate of substance k, $M_{k}$ is the molar mass of substance k, $H_{k}$ is the enthalpy per unit mass of substance k, the values of the enthalpy $H_{k}$ refer to Table 4 in reference [38], $T_{g}$ is the temperature at this location, λ is the thermal conductivity of the gas mixture, $\Gamma_{n}$ is the in situ density at surface position n, $Z_{k}(n)$ is the in situ fraction, $\sigma_{k}$ is the in situ occupancy number of substance k attached to n, and $s_{k}$ is the surface net reaction rate of substance k. In Equations (2) and (3), the absolute velocity $u_{k}$ of substance k is the sum of its diffusion velocity and convection velocity of substance k. Equation (4) describes the conservation of the surface material, where $\Gamma_{n}$ is the in situ density at surface position *n*, $Z_{k}(n)$ is the in situ fraction, $\sigma_{k}$ is the number of in situ occupations of substance k at the position n, and $s_{k}$ is the net surface reaction rate of surface substance k.

This paper primarily focuses on the film deposition process on the silicon wafer surface. In the aforementioned equations, the mass flux term and energy flux term are replaced by the corresponding gas surface flux matching terms. The revised equations are as follows [38]:

(5) $$\rho \omega_{k}u_{k}=r_{k}M_{k}\;(k=1,\ldots n),$$

(6) $$\rho u=\sum_{k=1}^{n}r_{k}M_{k},$$

(7) $$-k\nabla T_{g}+\sum_{k=1}^{n}\rho \omega_{k}H_{k}u_{k}=\sum_{k=1}r_{k}\omega_{k}H_{k}+Q,$$

Equation (5) is the mass flux conservation equation for substance k on the surface, Equation (6) is the mass flux equation for the gas phase, and Equation (7) is the energy conservation equation, where $r_{k}$ represents the net surface reaction rate of gas-phase substance k, and Q denotes the net heat exchange.

(8) $$Q{=\sigma }_{0}\varepsilon (T_{end}^{4}-T_{\mathrm{out}}^{4}).$$

$T_{end}$ is the temperature at the two extremities, $T_{out}$ is ambient temperature, $\sigma_{0}$ is Boltzmann constant, and ε is emissivity.

The net reaction rates of the above chemical equations can be expressed as follows:

(9) $$r_{j}=k_{j}^{f}\prod_{k\in react}c_{k}^{-v_{kj}}-k_{j}^{r}\prod_{k^{\prime }\in prod}c_{k^{\prime }}^{v_{k^{\prime }j}},$$

where $k_{j}^{f}$ represents the forward reaction coefficient of the chemical reaction, $k_{j}^{r}$ is the reverse reaction coefficient, $c_{k}$ is the concentration of substance k, and $v_{k}$ is the stoichiometric coefficient of substance k in the chemical reaction.

The forward reaction constant in Equation (9) can be expressed in the following form:

(10) $$k_{j}^{f}=A{\left(\frac{T}{T_{ref}}\right)}^{\beta }e^{\frac{-E}{R_{g}T}},$$

In this formula, the values of A, β, and E can be obtained from Table 1.

A, β, and **E** are all of the relevant parameters in Equation (10). Where **A** is pre-exponential factor, **β** is stoichiometric number and **E** is Arrhenius activation energy.

The reverse reaction constant can be determined through the following method:

(11) $$k_{j}^{r}=k_{j}^{f}/K_{eq},$$

where $K_{eq}$ represents the equilibrium constant of the chemical reaction, which can be calculated using the following equation:

(12) $$K_{eq}=e^{-\frac{\Delta H_{j}-T\Delta S_{j}}{R_{g}T}}.$$

In the equation, $\Delta H_{j}$ represents the enthalpy change of the chemical reaction, and $\Delta S_{j}$ represents the entropy change of the chemical reaction.

All gas-phase species and their reaction products will directly adsorb onto the silicon wafer surface, ultimately depositing as polysilicon films. The primary surface reactions that occur in this process are shown in Table 2.

The surface reaction rate (mol/m^2^·s) of polysilicon films can be calculated using Equation (13):

(13) $$r_{si}=\sum_{i=1}^{n}{k_{j}}^{f}\prod_{i\in react}{c_{i}}^{-v_{ij}},$$

The forward reaction coefficients for surface reactions can also be calculated using the following form:

(14) $$k_{j}^{f}=\left(\frac{\gamma_{i}}{1-\gamma_{i}/2}\right)\frac{\prod \sigma_{j}^{v_{ij}}}{{\left(\Gamma_{tot}\right)}^{m}}\left(\frac{1}{4}\right)\sqrt{\frac{8RT}{\pi M_{k}}},$$

where m represents the reaction order, $\Gamma_{hot}$ denotes the surface concentration (units: mol/m^2^), and R, T, $\gamma_{i}$, and $M_{k}$ represent the pre-exponential factor, temperature coefficient, gas constant, surface reaction temperature, viscosity coefficient, and relative molecular mass, respectively.

The net formation rate of polysilicon (in m/s) can be calculated using Equation (15):

(15) $$R_{si}=\frac{r_{si}\cdot M_{si}}{\rho_{si}}.$$

where $r_{si}$ represents the surface reaction rate of polysilicon (in mol/m^2^·s), $M_{si}$ is the molar mass of polysilicon, and $\rho_{si}$ is the density of polysilicon.

Si(b) represents the bulk Si and Si(s) denotes the surface Si species. A, β, and **E** are all relevant parameters in Equation (10). **A** is the pre-exponential factor, **β** is the stoichiometric number, and **E** is the Arrhenius activation energy. γ denotes the viscosity coefficient, which is the parameter of Equation (14).

## 3. Model Verification

The experimental substrates were n-type square monocrystalline silicon wafers with a side length of 182 mm, the silicon wafers were of a solar-grade silicon material with phosphorus used as a dopant. The polysilicon thin film LPCVD deposition time was about 1650 s, the pressure was 26 Pa, and the temperature was set as above; the thickness of the polysilicon film was measured using an ellipsometer. To increase the reliability of the data of the samples, the experimental data were obtained using the 5-point average method shown in Figure 2b. Therefore, by dividing the thickness of the film by the deposition time, we could obtain the deposition rate.

L_1_ was selected as the monitoring position, as shown in Figure 2a. The symmetric points were located 100 mm from the central point. Additionally, the simulation results regarding the polysilicon film thickness on L_1_ were selected for validation. The simulation data were taken at corresponding experimental silicon wafer positions.

**Figure 3:**
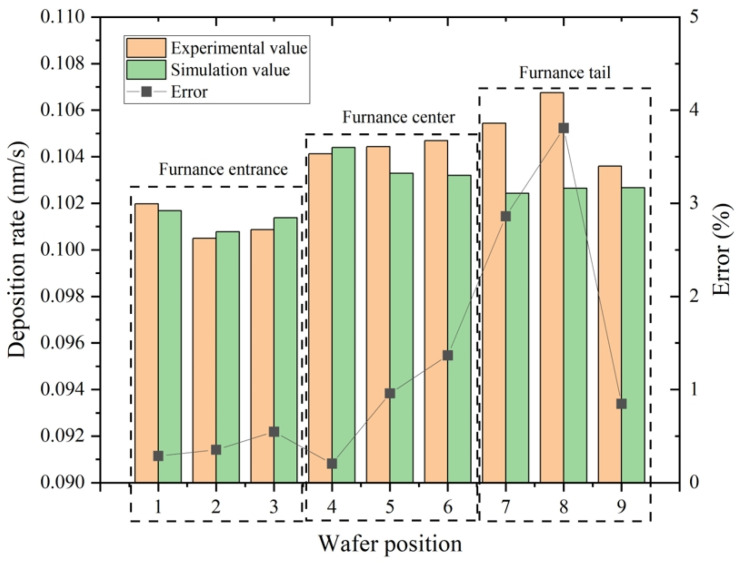
A comparison of experimental results and simulation value. The experimental data are averages of samples (refer to Figure 2’s annotation for the sample positions) The simulation results were taken from the silicon wafer positions on line L_1_ (Figure 2a), which are located at 1st, 120th, 240th, 481st, 600th, 720th, 961st, 1080th, and 1200th wafers, respectively.

The simulation verification was carried out, and the histogram is shown in Figure 3. It was found that the maximum error between the experimental data and simulation results was 4%, and the average value was about 1.5%. This indicates that the established simulation scheme is feasible.

## 4. Results and Discussion

To better describe the influence of precursors and the intermediates on polysilicon film deposition, the concept of the contribution rate of polysilicon film deposition was introduced by Equation (15).

(16) $$\theta_{k}=\frac{r_{k}}{R_{si}}.$$

where $\theta_{k}$ represents the contribution rate of the gas-phase substance to the polysilicon films, $r_{k}$ denotes the rate of the gas-phase substance k changing to the polysilicon films, and $R_{si}$ is the total deposition rate of the polysilicon films determined by Equation (15).

### 4.1. The Influence of Pressure

During the deposition process of the polysilicon film in a tubular LPCVD reactor, SiH_4_ undergoes gas-phase reactions and surface reactions. The contribution rates of gas-phase product SiH_4_ and intermediates Si_2_H_6_, H_2_SiSiH_2_, H_3_SiSiH, and SiH_2_ to the deposition process of polysilicon films at pressures of 20 Pa, 50 Pa, 100 Pa, and 200 Pa on L_1_ were summarized as a function of pressure.

**Figure 4:**
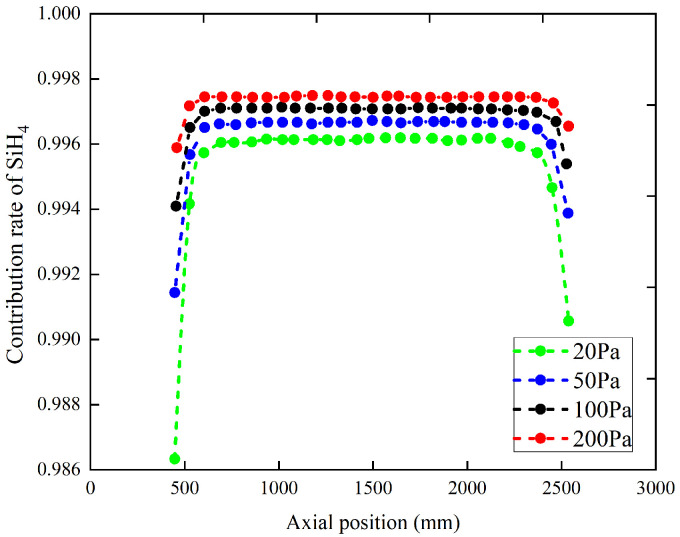
The influence of pressure on gas source SiH_4_ contribution rate distribution. The temperature is 884 K. The chamber pressures are 20 Pa, 50 Pa, 100 Pa, and 200 Pa, respectively.

As shown in Figure 4, the contribution rate of SiH_4_ to the deposition of the polysilicon film presents a distribution characteristic which is low at both ends and high in the middle. In contrast, the variation pattern of the contribution rate of four intermediates, Si_2_H_6_, H_2_SiSiH_2_, H_3_SiSiH, and SiH_2_ to the deposition of polysilicon films is the opposite, with high contributions at both ends and lower contributions in the middle. Moreover, there is a significant difference in the contribution rates of precursors and intermediates in terms of quantity. Due to the intermediates accumulated at the front and rear of the chamber whichdiffuse towards the center due to the concentration gradient, this also explains the axial contribution rate distribution characteristics of Si_2_H_6_, H_2_SiSiH_2_, H_3_SiSiH, and SiH_2_ gas-phase intermediates. In contrast, the molar fraction of SiH_4_ at the front and rear is relatively low, resulting in a lower contribution rate of SiH_4_ to the polysilicon film at both ends of the chamber.

**Figure 5:**
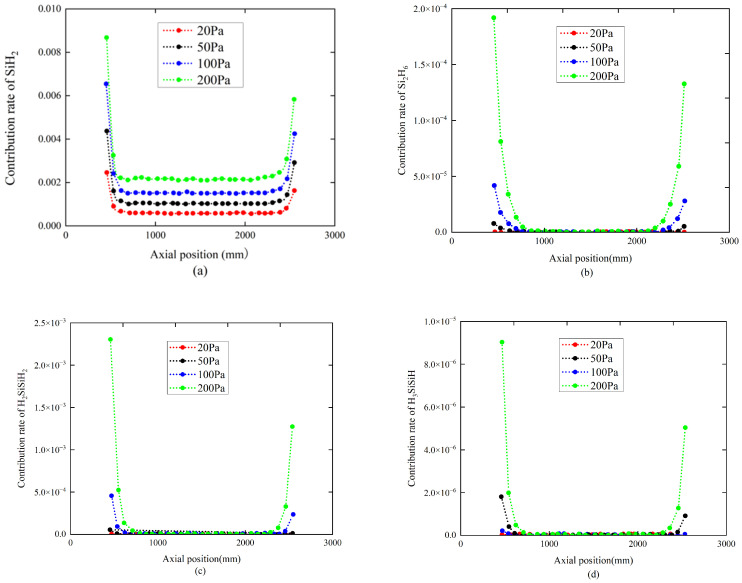
The influence of pressure on 4 types of intermediates’ contribution rate distribution. The temperature is 884 K. The chamber pressures are 20 Pa, 50 Pa, 100 Pa, and 200 Pa, respectively. (**a**) Gaseous SiH_2_; (**b**) gaseous Si_2_H_6_; (**c**) gaseous H_2_SiSiH_2_; and (**d**) gaseous H_3_SiSiH.

Generally, pressure significantly affects gas-phase reactions. As shown in Figure 4, with pressure increasing in the chamber, the contribution rate of SiH_4_ to the film decreases, while the contribution rates of the gas-phase, Si_2_H_6_, H_2_SiSiH_2_, H_3_SiSiH, and SiH_2_ exhibit an overall increasing trend. Higher pressure promotes the decomposition of more SiH_4_ molecules, leading to an increase in the molar fractions of intermediates. These intermediates also participate in the polysilicon film formation process, thereby reducing the contribution rate of SiH_4_. Additionally, the contribution rates of the three gas-phase intermediates, Si_2_H_6_, H_2_SiSiH_2_, and H_3_SiSiH, in the middle region of the reactor remain relatively constant as the pressure increases, as shown in Figure 5.

From Figure 4 and Figure 5, it is found that with further pressure increases in the chamber, the differences in the polysilicon film contribution rates in different regions become more pronounced. This is primarily because higher pressure reduces the diffusion capacity of gas-phase species, leading to a larger gradient in the contribution rates of gas-phase intermediates within the effective deposition area. Mainly due to different gas manifolds and radiative thermal exchange with the reactor doors which are colder than the tubular reactor walls, the result is a lower contribution rate at the two extremities.

From careful observation, it is found that when the chamber pressure is below 100 Pa, the overall contribution rate of SiH_4_ to the polysilicon film formation is above 99.6%. Therefore, when the chamber pressure is below 100 Pa, it is reasonable to ignore the contributions of the intermediates Si_2_H_6_, H_2_SiSiH_2_, H_3_SiSiH, and SiH_2_ to polysilicon film formation.

Based on the analysis results, establishing a simplified simulation model that ignores the effects of intermediates will greatly reduce resource consumption when simulating complex chemical reaction processes and save a significant amount of simulation time.

### 4.2. The Influence of Temperature

**Figure 6:**
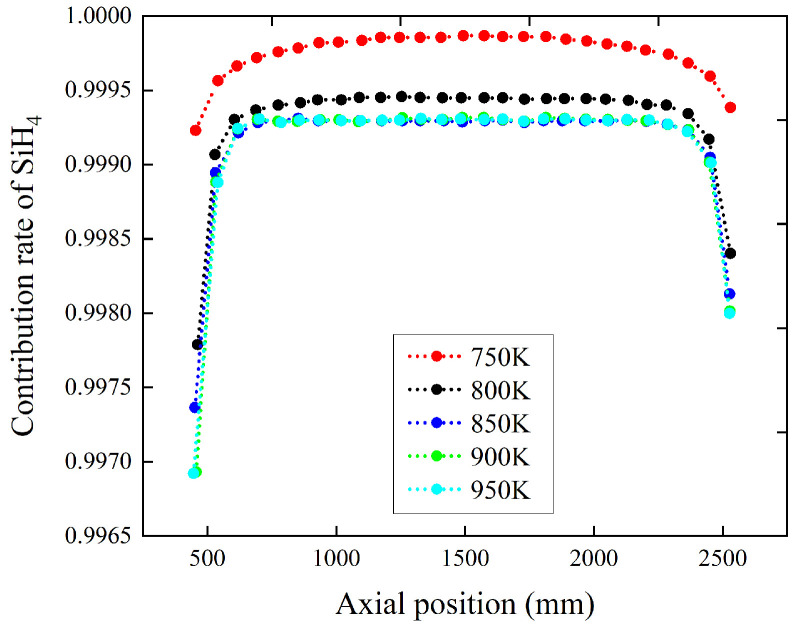
The influence of temperature on gas source SiH_4_ contribution rate distribution. The chamber pressure is 26 Pa. The temperatures are 750 K, 800 K, 850 K, 900 K, and 950 K, respectively.

**Figure 7:**
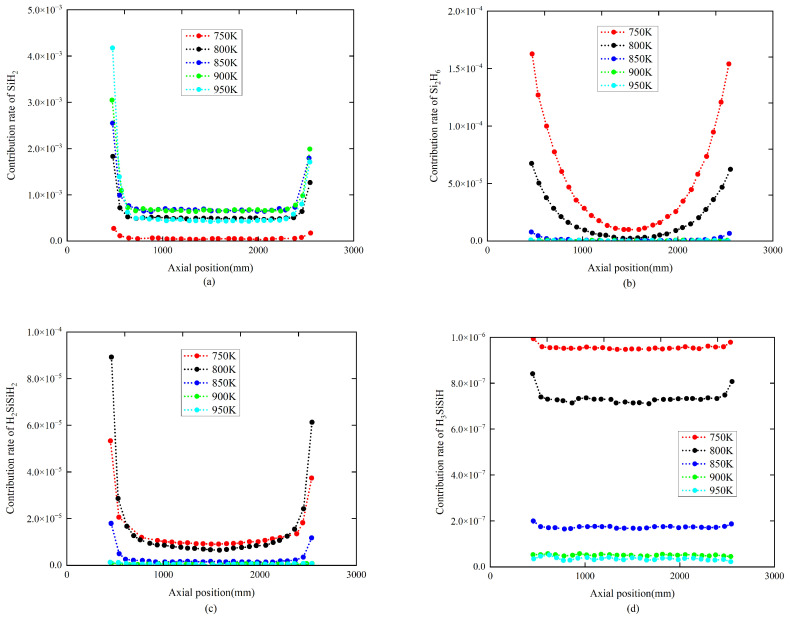
The influence of temperature on 4 types of intermediates’ contribution rate distribution. The chamber pressure is 26 Pa. The temperatures are 750 K, 800 K, 850 K, 900 K, and 950 K, respectively. (**a**) Gaseous SiH_2_; (**b**) gaseous Si_2_H_6_; (**c**) gaseous H_2_SiSiH_2_; and (**d**) gaseous H_3_SiSiH.

Keeping the pressure at 26 Pa, simulation experiments were conducted for five different temperatures of 750 K, 800 K, 850 K, 900 K, and 950 K. The simulation results indicate that increasing the temperature of the reaction chamber decreases the contribution rates of the four intermediates, SiH_2_, Si_2_H_6_, H_2_SiSiH_2_, and H_3_SiSiH, to the polysilicon films. When the chamber temperature reaches 850 K, further increasing the temperature results in almost no change in the contribution rates of these gas-phase intermediates. The simulation results are shown in Figure 6 and Figure 7. It is shown in Figure 6 that the contribution rates at the two extremities are more influenced by temperature. As can be seen from Figure 6, the contribution rates of different intermediates vary significantly. However, the magnitudes of these contribution rates are extremely small and can be neglected.

In TOPCon mass production processes, the deposition temperature of polysilicon films is generally above 850 K. According to the simulation results, under mass production conditions with process temperatures higher than 850 K, the simulation species can completely ignore the influence of the chamber temperature.

Notably, as seen in Figure 7, The contribution rates of intermediates in the two end regions vary significantly with temperature. Additionally, to improve the uniformity of film thickness, it is necessary to appropriately reduce the process temperature in this region. The synergistic adjustment of temperature and pressure should be the main process-guiding principle to further enhance photovoltaic conversion efficiency.

Clearly, under the conditions of pressure below 100 Pa and a temperature between 850 K and 950 K, the combined contribution rate of the four intermediates, SiH_2_, Si_2_H_6_, H_2_SiSiH_2_, and H_3_SiSiH, to the polysilicon films is less than 0.5%.

From the above-mentioned comprehensive analysis, temperature is an important fundamental condition for all chemical reactions. Additionally, utilizing refined temperature control methods can effectively adjust the temperature field. Employing porous, multi-position precursor supplementation is also a significant measure to regulate the uniformity of polysilicon film deposition. By adopting a temperature and pressure synergistic control strategy, the uniformity of polysilicon films can be greatly improved. This adjustment method can further improve the photoelectric conversion efficiency of photovoltaic cells.

In summary, considering temperature, pressure, and the use of SiH_4_ as a precursor in the process, the above analysis fully demonstrates that the impact of these four intermediates on the uniformity of film deposition can also be neglected.

## 5. The Proposal of the Simplified Reaction Mechanism Model

Based on the research conclusions of reference [38], the COMSOL chemical reaction module, in which there are four kinds of gas-phase reactions (in Table 1) and six kinds of surface reactions (in Table 2), has been established in the paper. There are four intermediates in the gas-phase reaction process, and six types of multi-step surface reactions occur between methane and the four intermediates. In Table 2, the equations in the first two lines are a set of self-consistent reaction equations that can be independent of other reaction equations. Moreover, after carefully analyzing our simulation results, we found that this step dominates the growth rate of polysilicon thin films, with a polysilicon contribution rate of no less than 99.5%. This result fully demonstrates that this group of reactions controls the growth rate of polysilicon thin films. Based on this, we propose that the complex four-kind gas-phase reactions and their symbiotic four-kind surface reactions can be ignored for the simulation of the chemical reaction process. The refined and simplified module establishment greatly simplifies the real reaction process and reduces the number of iterative steps in the simulation loop, thereby saving expensive simulation resources and making it possible to establish a three-dimensional simulation of a large-sized LPCVD polysilicon thin film deposition system.

Returning to the core issues of the contribution rate of polysilicon films, our simulation results clearly demonstrate that the impact of the intermediates, SiH_2_, Si_2_H_6_, H_2_SiSiH_2_, and H_3_SiSiH, on the polysilicon film deposition can be neglected. Therefore, the polysilicon film deposition reaction process can be simplified as follows:

(17) $${\mathrm{SiH}}_{4}{+2\mathrm{Si}(s)=>\mathrm{Si}(b)+2\mathrm{SiH}(s)+H}_{2}$$

(18) $${2\mathrm{SiH}(s)=>2\mathrm{Si}(s)+H}_{2}$$

Equations (17) and (18) are important conclusions from reference [38]. As a result of our efforts, we believe that the customary chemical reaction module in the COMSOL Multiphysics software can be replaced with Equations (17) and (18). This replacement will greatly simplify the simulation process and make the model much clearer.

In the refined simplified module, intentionally ignoring the complex gas-phase intermediates does not harm the accuracy of the film contribution rate, but it can significantly improve simulation efficiency. Replacing the actual reaction process with the refined simplified module releases more computing power. Under the refined chemical reaction process model premise, further construction of an efficient simulation model containing three-dimensional spatial information can obtain more diverse parameters of film uniformity. This is precisely the direction we aim to pursue in the future.

## 6. Conclusions

This article refines a multi-physics field coupling simulation model for the deposition of polysilicon films on a large-sized tubular LPCVD reactors and explores the contribution rate of precursor material SiH_4_ and intermediates to film deposition.

The simulation results show that it is feasible to neglect the impact of intermediates on the deposition contribution rate of polysilicon films. Under the condition where the chamber temperature is not less than 850 K, the contribution rate of SiH_4_ to the deposition of polysilicon films remains essentially unchanged. The contribution rate of SiH_4_ to the deposition of polysilicon films exceeds 99.6%. The contribution rates of the intermediates SiH_2_, Si_2_H_6_, H_2_SiSiH_2_, and H_3_SiSiH to the deposition of polysilicon films are extremely low. Therefore, the impact of these four intermediates on the uniformity of film deposition can also be ignored.

A refined chemical reaction module neglecting the influence of intermediates is proposed. With this new module, massive iterative simulation steps for real chemical reaction processes can be saved, making it possible to establish a three-dimensional simulation platform of a large-sized tubular LPCVD polysilicon thin film deposition system using the COMSOL Multiphysics software.

## Figures and Tables

**Table 1:** Primary homogeneous gas-phase chemical reactions for polysilicon from SiH_4_ pyrolysis for 800–950 K [38].

Reaction Equation	A (mol, cm^3^, s)	β (-)	E (J/mol)
${\mathrm{SiH}}_{4}⇌{\mathrm{SiH}}_{2}{+H}_{2}$	1.28 × 10^10^	0	215,800
${\mathrm{SiH}}_{4}{+\mathrm{SiH}}_{2}⇌{\mathrm{Si}}_{2}H_{6}$	3.53 × 10^10^	0	163,300
${\mathrm{Si}}_{2}H_{6}⇌H_{2}{+H}_{3}\mathrm{SiSiH}$	9.68 × 10^10^	0	180,600
$H_{3}\mathrm{SiSiH}⇌H_{2}{\mathrm{SiSiH}}_{2}$	6.02 × 10^10^	0	4200

**Table 2:** Primary surface chemical reactions for polysilicon from SiH_4_ pyrolysis for 800–950 K [38,39].

Reaction Equation	A (mol, cm^3^, s)	β (-)	E (J/mol)
${\mathrm{SiH}}_{4}{+2\mathrm{Si}(s)=>\mathrm{Si}(b)+2\mathrm{SiH}(s)+H}_{2}$	8.39 × 10^16^	0	37,450
${2\mathrm{SiH}(s)=>2\mathrm{Si}(s)+H}_{2}$	1.75 × 10^16^	0	47,000
${\mathrm{Si}}_{2}H_{6}{+2\mathrm{Si}(s)=>2\mathrm{Si}(b)+2\mathrm{SiH}(s)+2H}_{2}$	8.39 × 10^16^	0	37,450
$H_{2}{\mathrm{SiSiH}}_{2}{\;=>2\mathrm{Si}(b)+2H}_{2}$		γ=1	
$H_{3}{\mathrm{SiSiH}\;=>2\mathrm{Si}(b)+2H}_{2}$		γ=1	
${\mathrm{SiH}}_{2}{\;=>\mathrm{Si}(b)+H}_{2}$		γ=1	

## Data Availability

The original contributions presented in the study are included in the article, further inquiries can be directed to the corresponding authors.

## References

[1] Padhamnath P., Nandakumar N., Kitz B.J., Balaji N., Naval M.J., Shanmugam V., Duttagupta S. (2018). High-quality doped polysilicon using low-pressure chemical vapor deposition (LPCVD). Energy Procedia.

[2] Ma Z.Q., Wang Y.L., Lan Z.X., Zhao L., Xu F., Xu J. (2022). On the presence of oxy phosphorus bonds in TOPCon solar cell poly silicon films. Sol. Energy Mater. Sol. Cells.

[3] Trainor M. (1992). Studies of low-pressure chemical vapor deposition (LPCVD) of polysilicon (chemical vapor deposition). eLIBRARY.RU.

[4] Zeng Y., Tong H., Quan C., Cai L., Yang Z., Chen K., Yuan Z., Wu C.H., Yan B., Gao P. (2017). Theoretical exploration towards high-efficiency tunnel oxide passivated carrier-selective contacts (TOPCon) solar cells. Sol. Energy.

[5] Zhang Z., Zeng Y., Jiang C.S., Huang Y., Liao M., Tong H., Al-Jassim M., Gao P., Shou C., Zhou X. (2018). Carrier transport through the ultrathin silicon-oxide layer in tunnel oxide passivated contact (TOPCon) c-Si solar cells. Sol. Energy Mater. Sol. Cells.

[6] Zhou J., Lv B., Liang H., Wen Z. (2023). Simulation and optimization of polysilicon thin film deposition in a 3000 mm tubular LPCVD reactor. Sol. Energy.

[7] Truong T.N., Yan D., Nguyen C.P.T., Kho T., Guthrey H., Seidel J., Al-Jassim M., Cuevas A., Macdonald D., Ngu-yen H.T. (2021). Morphology, microstructure, and doping behavior: A comparison between different deposition methods for poly-Si/SiOx passivating contacts. Prog. Photovolt. Res. Appl..

[8] Chen Y., Meng X., Fan J., Deng M., Ye H., Qian C., Zhang P., Xing G., Yu J. (2023). Effects of LPCVD-deposited thin intrinsic silicon films on the performance of boron-doped polysilicon passivating contacts. Sol. Energy.

[9] Fırat M., Radhakrishnan H.S., Payo M.R., Choulat P., Badran H., van der Heide A., Govaerts J., Duerinckx F., Tous L., Hajjiah A. (2022). Large-area bifacial n-TOPCon solar cells with in situ phosphorus-doped LPCVD poly-Si passivating contacts. Sol. Energy Mater. Sol. Cells.

[10] Cao Y., Zhou J., Ren Y., Xu W., Liu W., Cai X., Zhao B. (2020). Study on effect of process and structure parameters on SiNxHy growth by in-line PECVD. Sol. Energy.

[11] Zhou J., Huang J., Liao J., Guo Y., Zhao Z., Liang H. (2021). Multi-field simulation and optimization of SiNx: H thin-film deposition by large-sized tubular LF-PECVD. Sol. Energy.

[12] Zhou J., Xu W., Chen T. (2021). An investigation on improving the homogeneity of plasma generated by linear microwave plasma source with a length of 1550 mm. Plasma Sci. Technol..

[13] Zhou J., Liao J., Huang J., Chen T., Lv B., Peng Y. (2022). Effects of process parameters and chamber structure on plasma uniformity in a large-area capacitively coupled discharge. Vacuum.

[14] Coltrin M.E., Kee R.J., Miller J.A. (1984). A mathematical model of the coupled fluid mechanics and chemical kinetics in a chemical vapor deposition reactor. J. Electrochem. Soc..

[15] Coltrin M.E., Kee R.J., Evans G.H. (1989). A mathematical model of the fluid mechanics and gas-phase chemistry in a rotating disk chemical vapor deposition reactor. J. Electrochem. Soc..

[16] Coltrin M.E., Kee R.J., Miller J.A. (1986). A mathematical model of silicon chemical vapor deposition: Further refinements and the effects of thermal diffusion. J. Electrochem. Soc..

[17] Kinoshita S., Takagi S., Kai T., Shiozawa J., Maki K. (2005). Multiscale Analysis of Silicon Low-Pressure Chemical Vapor Deposi-tion. Jpn. J. Appl. Phys..

[18] Kleijn C.R. (1991). A mathematical model of the hydrodynamics and gas-phase reactions in silicon LPCVD in a single-wafer reactor. J. Electrochem. Soc..

[19] Shimizu R., Ogino M., Sugiyama M., Shimogaki Y. (2007). Predictive model extraction for polysilicon low-pressure chemical vapor deposition in a commercial scale reactor. J. Electrochem. Soc..

[20] Houf W.G., Grcar J.F., Breiland W.G. (1993). A model for low pressure chemical vapor deposition in a hot-wall tubular reactor. Mater. Sci. Eng. B.

[21] Ho P., Coltrin M.E., Breiland W.G. (1994). Laser-induced fluorescence measurements and kinetic analysis of Si atom formation in a rotating disk chemical vapor deposition reactor. J. Phys. Chemi..

[22] Kleijn C.R. (2000). Computational modeling of transport phenomena and detailed chemistry in chemical vapor deposition—A benchmark solution. Thin Solid Films.

[23] Kleijn C.R., Van Der Meer T.H., Hoogendoorn C.J. (1989). A mathematical model for LPCVD in a single wafer reactor. J. Electrochem. Soc..

[24] Peng Y., Feng T., Zhou J. (2022). Effect of power ratio of side/top heaters on the performance and growth of multi-crystalline silicon ingots. Mater. Lett..

[25] (2024). TOPCon Will Remain Mainstream in the Next Five Years. https://mp.weixin.qq.com/s/IfUY4QvdZg_hbdbAb5Djcg.

[26] Padhamnath P., Nampalli N., Nandakumar N., Buatis J.K., Naval M.J., Aberle A.G., Duttagupta S. (2020). Optoelectrical properties of high-performance low-pressure chemical vapor deposited phosphorus-doped polysilicon layers for passivating con-tact solar cells. Thin Solid Films.

[27] Temple-Boyer P., Rousset B., Scheid E. (2010). Influences of deposition and crystallization kinetics on the properties of silicon films deposited by low-pressure chemical vapor deposition from SiH_4_ and diSiH_4_. Thin Solid Films.

[28] Ghosh D.K., Bose S., Das G., Acharyya S., Nandi A., Mukhopadhyay S., Sengupta A. (2022). Fundamentals, present status and future perspective of TOPCon solar cells: A comprehensive review. Surf. Interfaces.

[29] Guan K., Gao Y., Zeng Q., Luan X., Zhang Y., Cheng L., Wu J., Lu Z. (2020). Numerical modeling of SiC by low-pressure chemical vapor deposition from methyltrichlorosilane. Chin. J. Chem. Eng..

[30] Mondal A., Yadav M.K., Bag A. (2020). Transition from thin film to nanostructure in low pressure chemical vapor deposition growth of β-Ga_2_O_3_: Impact of metal gallium source. Thin Solid Films.

[31] Kanneboina V. (2022). Detailed review on c-Si/a-Si: H heterojunction solar cells in perspective of experimental and simulation. Mi-Croelectron. Eng..

[32] Satpathy R., Pamuru V. (2021). Making of crystalline silicon solar cells. Elsevier eBooks.

[33] Weerts W.L.M., De Croon M.H.J.M., Marin G.B. (1996). Low pressure chemical vapor deposition of polysilicon: Validation and assessment of reactor models. Chem. Eng. Sci..

[34] Azzaro C., Duverneuil P., Couderc J.P. (1992). Thermal and kinetic modelling of low-pressure chemical vapor deposition hot-wall tubular reactors. Chem. Eng. Sci..

[35] Zhou Y., Tao K., Liu A., Jia R., Bao J., Sun Y., Yang S., Wang Q., Zhang Q., Yang S. (2020). The impacts of LPCVD wrap-around on the performance of n-type tunnel oxide passivated contact c-Si solar cell. Curr. Appl. Phys..

[36] Wang Q., Wu W., Yuan N., Li Y., Zhang Y., Ding J. (2020). Influence of SiOx film thickness on electrical performance and efficiency of TOPCon solar cells. Sol. Energy Mater. Sol. Cells.

[37] COMSOL (2020). CFD Module User’s Guide, COMSOL Multiphysics® v. 5.6.

[38] Weerts W.L.M., de Croon M.H.J.M., Marin G.B. (1998). The kinetics of the low-pressure chemical vapor deposition of poly-crystalline silicon from SiH_4_. J. Electrochem. Soc..

[39] Nijhawan S., McMurry P.H., Swihart M.T., Suh S.M., Girshick S.L., Campbell S.A., Brockmann J.E. (2003). An experimental and numerical study of particle nucleation and growth during low-pressure thermal decomposition of SiH_4_. J. Aerosol Sci..

